# Snapshot of the Distribution and Biology of Alien Jellyfish *Cassiopea andromeda* (Forsskål, 1775) in a Mediterranean Touristic Harbour

**DOI:** 10.3390/biology11020319

**Published:** 2022-02-16

**Authors:** Tiziana Cillari, Alessandro Allegra, Daniela Berto, Mar Bosch-Belmar, Manuela Falautano, Teresa Maggio, Giacomo Milisenda, Patrizia Perzia, Federico Rampazzo, Mauro Sinopoli, Luca Castriota

**Affiliations:** 1 Italian Institute for Environmental Protection and Research, Department for the Monitoring and Protection of the Environment and for the Conservation of Biodiversity, Unit for Conservation Management and Sustainable Use of Fish and Marine Resources, Lungomare Cristoforo Colombo 4521 (Ex Complesso Roosevelt), Località Addaura, 90149 Palermo, Italy; tiziana.cillari@isprambiente.it (T.C.); manuela.falautano@isprambiente.it (M.F.); teresa.maggio@isprambiente.it (T.M.); patrizia.perzia@isprambiente.it (P.P.); luca.castriota@isprambiente.it (L.C.); 2 GRAM Gruppo di Ricerca Applicata al Mare Soc. Coop., 90100 Palermo, Italy; a22allegra@gmail.com; 3 Italian Institute for Environmental Protection and Research, Department for the Monitoring and Protection of the Environment and for the Conservation of Biodiversity, Unit for Marine Waters and Ecosystems Monitoring and Characterisation, Località Brondolo, 30015 Chioggia, Italy; daniela.berto@isprambiente.it (D.B.); federico.rampazzo@isprambiente.it (F.R.); 4 University of Palermo, Department of Earth and Marine Sciences (DiSTeM), Via Archirafi 22, 90123 Palermo, Italy; mariadelmar.boschbelmar@unipa.it; 5 Stazione Zoologica Anton Dohrn, Department of Integrative Marine Ecology (EMI), Sicily Marine Centre, Lungomare Cristoforo Colombo 4521 (Ex Complesso Roosevelt), Località Addaura, 90149 Palermo, Italy; giacomo.milisenda@szn.it

**Keywords:** non-indigenous species, upside-down jellyfish, Megabenthos Underwater Video, species distribution, stable isotopes, mixotrophic behavior

## Abstract

**Simple Summary:**

Alien species are an important cause of biodiversity loss and changes to ecosystems. Harbors are hotspots for the introduction of these species, and, usually, the impacts and pathways of invasion of the host populations are poorly known. Since 2014, an alien jellyfish, *Cassiopea andromeda*, coming from the Red Sea, has invaded a Mediterranean touristic harbor and established a population there. In this study, the distribution and trophic behavior of *C. andromeda* were investigated to improve knowledge on this species within the Mediterranean. The preliminary results highlight and confirm that *C. andromeda* is a perfect invader thanks to its nutritional strategy and capacity to adapt to heavily anthropized areas. Therefore, its potential impact on the local biodiversity and thus on the ecosystem’s structure and functioning is worth considering.

**Abstract:**

Harbors are hotspots for the introduction of alien species, and, usually, investigations on their host populations help fill the knowledge gap in their pathways of invasion and in their impacts on marine biodiversity and ecosystems. In 2014, the upside-down alien jellyfish *Cassiopea andromeda* invaded a Mediterranean touristic harbor (“Cala”), and its abundance has since increased over time. In the present study, the distribution and trophic behavior of *C. andromeda* in Cala were investigated for the years 2017–2018 through visual sampling, and GIS-based statistical and stable isotope analyses. Since Cala is a hard-to-reach area (with many anchor cables and boats), Megabenthos Underwater Video was used to count the number and estimate the size of jellyfishes. The variations in size throughout the study period suggest that the population of *C. andromeda* is quite established in Cala at depths lower than 7.5 m. The ranges of the environmental parameters recorded (temperature, salinity, and transparency) were consistent with the ideal conditions for maintaining a *Cassiopea* population, but they did not seem to influence aggregation. Additionally, the carbon and nitrogen isotopic signatures studied highlight the mixotrophic behavior of this species. These preliminary results confirm the capacity of *C. andromeda* to live and reproduce in heavily anthropized areas.

## 1. Introduction

The presence of non-indigenous species (NIS), also called alien species, is considered an important cause of biodiversity loss and changes in an ecosystem [1]. In the Mediterranean Sea, NIS can also be introduced via maritime traffic, by means of ballast waters and hull fouling. The diffusion of NIS has also been correlated with climate changes, which allows tropical and subtropical species to expand their distribution to other habitats [2,3,4]. For these reasons, harbors are hotspots for the introduction of NIS, and, usually, the host populations need to be studied to fill the knowledge gap in their pathways of invasion and in their impacts on the surrounding ecosystems. In 2014, the Lessepsian upside-down jellyfish *Cassiopea andromeda* (Forsskål, 1775) (Cnidaria, Rhizostomeae) invaded a touristic harbor of Palermo (Sicily, Italy) named Cala. Over the years, its abundance within Cala has increased [5].

**Figure 1:**
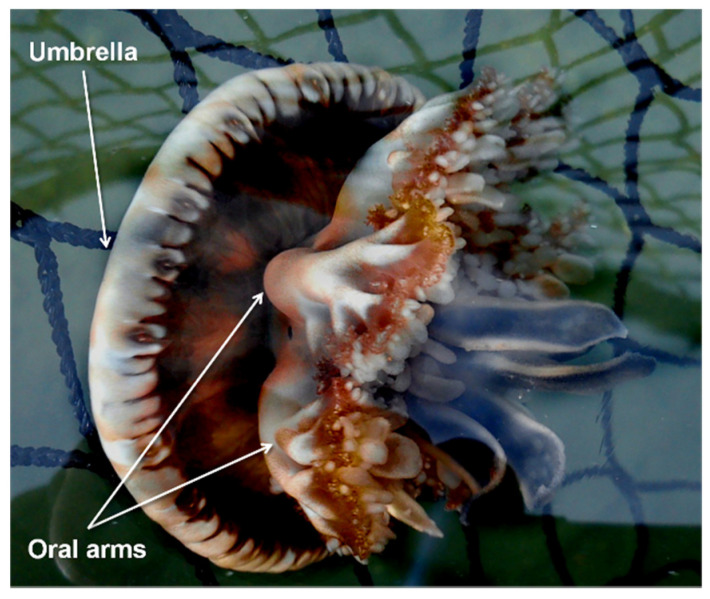
*Cassiopea andromeda* (Forsskål, 1775).

*C. andromeda* (Figure 1) entered the Mediterranean from the Red Sea through the Suez Canal [5,6] and colonized several areas of the basin. This species is an epibenthic scyphozoan with a maximum umbrella diameter of about 30 cm commonly found in tropical and subtropical shallow coastal ecosystems such as mangroves, estuaries, and sandy mudflats. This species has a metagenetic cycle with the following phases: planula, benthic polyp, ephyra, and adult medusa [7].

Contrary to jellyfish with pelagic behaviors, these benthic jellyfish prefer habitats with less water movements [8,9,10]. They often lay down on their umbrella, exposing their oral arms to the sun. Similar to other Rhizostomeae jellyfish, they frequently exhibit a symbiotic relationship with dinoflagellates, such as *Symbiodinium* spp., which are present in the tentacular tissue on their oral arms [11]. This relationship allows the jellyfish species to feed via direct predation and through photosynthesis by the zooxanthellae [12,13]; this mixed feeding of the jellyfish is called mixotrophy (concurrent autotrophy and heterotrophy).

In this symbiotic interaction, the carbon needed for the basal metabolism of the host is provided by the photosynthetic process [14,15,16,17], whereas nitrogen is taken up from the environment through the digestive processes [12,13]. However, this amount of nutrient intake is not fixed but rather depends on the species and the environments in which they grow (see, e.g., [16,17,18,19,20]). As with other symbiotic cnidarians, *Cassiopea* jellyfish may acquire dissolved nutrients from the surrounding environment to meet the energetic needs of their photosynthetic partners [21], while zooxanthellae may provide much of the carbon requirements to the host, which are critical to the metamorphosis of ephyrae and to the survival of the jellyfish [8]. Stable carbon and nitrogen isotopes might be valuable indicators of the relative importance between the autotrophy and heterotrophy pathways [22,23,24]. Subsequent studies have found that several factors such as inorganic nitrogen uptake [25], terrestrial nitrogen loads [26], eutrophication [26,27,28,29], zooxanthellae population dynamics [30], light [31], and bleaching [32] may affect the nitrogen isotopic (δ^15^N) signature of mixotrophic coral organisms with zooxanthellae symbionts.

Some authors have demonstrated the success of a mutualistic interaction with *C. andromeda* under stressful environmental conditions in shallow waters, i.e., high temperatures, high levels of irradiation, eutrophic conditions, and changes in salinity [33]. These life history traits also permitted this species to become an invader in the Mediterranean Sea, where, being one of the earliest Lessepsian migrants, *C. andromeda* has spread, reaching the western Mar Menor in Spain, being randomly spotted in the Levant Sea, Aegean Sea, and Strait of Sicily [6,34,35,36]. Mediterranean *C. andromeda* populations form short-term outbreaks up to 20 individuals m^−2^ in semi-enclosed human-impacted coastal systems with eutrophic waters and low hydrodynamics [5]. Stoner et al. [8] observed that *Cassiopea* spp. populations were significantly denser, and that individuals from these populations were larger in areas with high human population densities (a proxy of nutrient enrichment) with respect to more natural sites. Equally, The et al. [37] reported high densities of *Cassiopea* jellyfish within shrimp farms where environmental conditions were stable and the concentrations of nutrients and organic matter were high.

Although knowledge about the biology and ecology of *C. andromeda* in its native distribution areas is available, little is known about its invasive behavior in the Mediterranean Sea [38]. Given the growing interest in invasive alien species and in their management in the Mediterranean, recently, the capability of *C. andromeda* to tolerate stressful/changing environments (such as harbor areas) has been studied [38]. The authors of that study showed that this jellyfish species has the ability to adapt to sudden changes in light exposure from eutrophic to meso-oligotrophic waters and to withstand different light conditions, thus suggesting that this species can colonize a wide range of shallow water environments. These characteristics make it a potential successful invader in the Mediterranean Sea.

In this paper, a preliminary investigation on a Mediterranean population of *C. andromeda* (Palermo, Italy) was carried out through visual sampling and specimen collection. The data were then organized and processed to provide insights into the distribution and trophic behavior of this species within the study area.

## 2. Materials and Methods

*Cassiopea andromeda* were studied using a visual census and by direct collection.

### 2.1. Study Area

**Figure 2:**
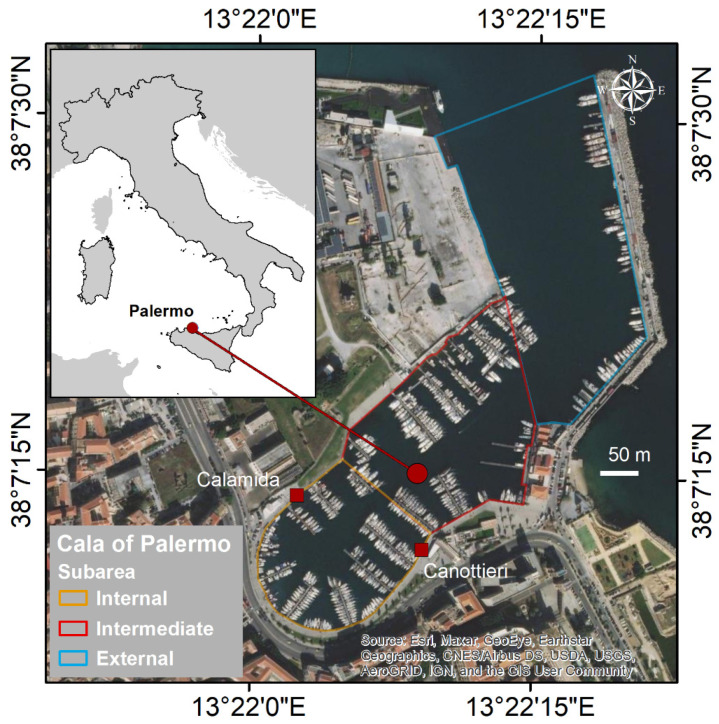
Study area: Cala of Palermo.

Cala is a curvilinear boat basin for pleasure crafts reachable from the mouth of the port of Palermo (Figure 2); it has an area of 49,000 m^2^, and an average depth of 7 m [39]. The sea bottom is predominantly made up of clay and is subject to rearrangement due to shipping activities and possible inputs from the land (e.g., run-off water and old underground channels). This area is characterized by the presence of many piers located very close to each other, mainly during the tourist season, that can accommodate up to 370 boats [39]. This proximity results in a large presence of ropes, anchor cables, and boats that make the sea bottom difficult to access for classical visual observations.

### 2.2. Visual Sampling Activities

The visual observations were carried out using Megabenthos Underwater Video (MUV), which was designed to overcome visual sampling difficulties in harbor areas [40].

**Figure 3:**
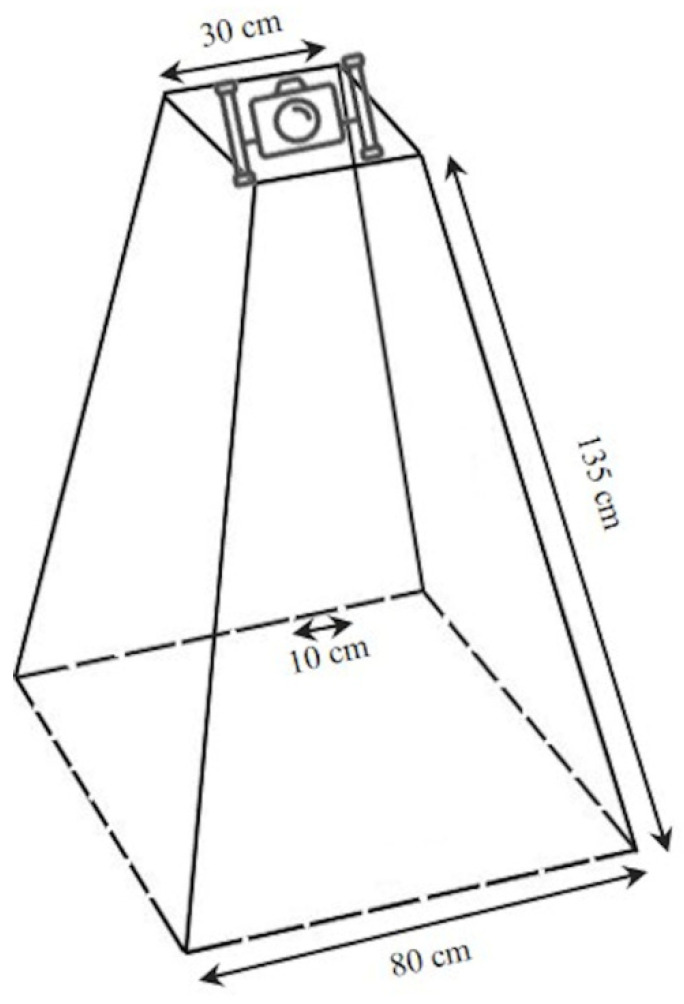
Technical features of the Megabenthos Underwater Video.

This device consists of stainless-steel tubes with 5 mm diameters and has a truncated square pyramid shape with a base side of 80 cm (unit of sampling area = 0.64 m^2^), and an oblique side that is 135 cm long. An underwater video camera is located on the top. The base sides are marked at 10 cm intervals to be used as a tool for estimating the size of individuals (Figure 3). Its base is lead ballasted to maintain an upright position during hauling of the device to the seafloor; a metered rope is used to record depth.

MUV was used in Cala to collect data on the density and size of *C. andromeda* individuals. No a priori sampling design was planned; the MUV hauls were carried out at random sites within Cala on four dates (June 2017, November 2017, February 2018, and April 2018). The observations in each sampling data were considered independent from each other. The device was hauled from a small boat to the sea bottom, recording the haul using the cam. In addition to depth (m), at each sampling site, temperature (°C) and salinity along the water column were recorded using a portable meter (VWR EC300), and water transparency (m) was recorded with a Secchi disc. The latter parameter was reported as a percentage ratio between water transparency (m) and depth (m).

### 2.3. Specimen Collection and Sample Preparation

Ten live *C. andromeda* specimens were collected in the quay facing two sites of Cala (five individuals in front of “Calamida” and five in front of “Canottieri”—Figure 2) using a hand net for the subsequent stable isotope analysis (SI). The specimens were placed in a 50 L tank, and immediately after sampling (~2 h), the jellyfish were brought to the laboratory to proceed with the morphometric and morphological analysis. The gonadal tissue was dissected from the somatic tissue for each specimen, and each was separately frozen in liquid nitrogen for subsequent trophic biomarker analyses and stored at −80 °C.

To obtain the particulate organic matter for SI analysis, 3 L of seawater (sampled from each site at a depth of 1 m) was pre-filtered (200 μm) and then filtered under vacuum through one pre-ashed (4 h at 500 °C) Whatman GF/F glass fiber filter (47 mm).

The sediment was collected using sediment traps to analyze its role in the trophic web. Each trap consisted of two plastic bottles with a funnel (15 cm diameter), left at the bottom of the two sampling sites for 7 days. After 7 days, the traps were closed, removed from the bottom, and brought to the laboratory. The water and sediment contained in the trap were filtered under vacuum through pre-ashed (4 h at 500 °C) Whatman GF/F glass fiber filters.

At the same time as sampling of the jellyfish, POM, and sediments, other benthic organisms were sampled to obtain a clearer reference of the trophic positioning of jellyfish within Cala. These organisms were sorted, stored separately in falcon or labeled plastic bags, and stored at −80 °C. After storage, all samples were freeze-dried and ground to a fine powder.

### 2.4. Data Analysis

#### 2.4.1. Visual Data Analysis

The data from the MUV observations were used to preliminarily investigate the *C. andromeda* size composition and distribution in the study area.

The videos from each sampling were analyzed visually on a PC workstation. For each video, the number of individuals of *C. andromeda* per unit of sampling area (m^2^) was recorded, counting the number of individuals for which their umbrella center was inside the unit area. The umbrella’s diameter was also measured. Three size classes (5–10 cm, 10–15 cm, and 15–20 cm) and four depth ranges (0–3 m, 3–6 m, 6–9 m, and 9–12 m) were defined. The average (±standard error) number of individuals belonging to each size class per unit area (average density, ind m^−2^) was calculated per sampling date and depth range. The variations in density among size classes were compared using PERMANOVA [41] with two fixed factors: sampling date and depth range. The analyses were based on a Euclidean distance matrix generated using square-root-transformed data and 999 permutations. A *p* value < 0.05 was considered statistically significant. The statistical analyses were run using the Primer 6 PERMANOVA+ software package (Plymouth Marine Laboratory). Finally, Cala was divided into three sub-areas (internal, intermediate, and external), and for each one of these, the average densities of individuals per size class and per depth range were calculated and displayed on maps using ArcGIS 10.3 software. PERMANOVA was also performed to detect differences in the densities of each size class: at each depth range among sampling dates; among sub-areas; and at each depth range among sub-areas.

#### 2.4.2. Spatial and Temporal Distribution Analysis

The data from the MUV observations were analyzed using ArcGIS 10.3 ESRI and its Spatial Statistics tools (Measuring Geographic Distributions, Analysing Patterns, and Mapping Clusters toolsets). GIS-based spatial statistics analysis is useful in the study of species distribution [42] and alien species distribution and spread [43,44]. These analyses were carried out to model the spatial and temporal distributions, aggregation patterns, and spatial structure of *C. andromeda* in the study area; these analyses allowed obtaining information related to the areas of concentration for this species on different sampling dates: the shape of the distributions (dispersed/compact or elongated); the distribution patterns (clustered, dispersed, or random); and their dependency on some environmental parameters such as temperature, salinity, and transparency.

To describe and summarize the key distribution characteristics and to track their changes over time and space, the indicators of central tendency (as a median center), species spatial dispersion, directional dispersion, and directional trend were calculated. These indicator values track the changes in distribution over time and space and allow for a comparison of the distributions of occurrences between sampling dates and size classes [45,46]. The spatial dispersion shows whether the distribution is concentrated or dispersed around the geometric mean center: the higher the standard distance (i.e., the radius of the generated circumference), the more dispersed the distribution [47]. The directional dispersion and directional trends allow us to visualize the shape of the distribution in the X and Y directions (Standard Deviational Ellipse) and the orientation of the distribution (the long-axis rotation of an ellipse measured clockwise from noon) [47]. The spatial dispersion and directional dispersion were calculated with a one-standard-deviation polygon that covers approximately 68% of the features. After data collection, a comparison of the distribution of *C. andromeda* specimens between the internal and intermediate sub-areas in June and November, when both sub-areas were investigated, was conducted. The distribution of the size classes (with the exception of the size class 15–20 cm, which had a number of observations that were insufficient for the analyses) was also analyzed in February, when the observations were carried out only in the internal sub-area. All data from April were excluded as the number of specimens recorded was not sufficient for the analyses.

In this study, the overall aggregation patterns and spatial structures of the distribution of *C. andromeda* in the area in relation to the environmental parameters temperature (T), salinity (S), and water transparency (Tr) were also evaluated. To assess if the species distribution pattern was clustered, dispersed, or random, spatial autocorrelation through Global Moran’s I (GMI) was carried out (cut-off distance of 60 m). The GMI method was performed simultaneously for the recorded position and for an associated attribute (ESRI ArcGIS, 2011). GMI ranges from −1 to +1, and when the z-score is statistically significant (*p* < 0.01), a positive or negative GMI indicates a spatially clustered or dispersed dataset, respectively [47]. Getis–Ord Gi* hot spot analyses (GOG) were carried out (cut-off distance of 60 m) to evaluate if the spatial structure featured statistically significant spatial clusters. Statistically significant z-scores (*p* < 0.05) indicate spatial clustering of high or low feature values (hot spots or cold spots, respectively). The higher (or lower) the z-score, the more (or less) intense the clustering. A z-score near zero indicates no apparent spatial clustering [47].

#### 2.4.3. Stable Isotope

Stable isotope analysis (SIA) is useful in explaining the role and effects of alien species in the food web [48]. In this research, stable carbon (δ^13^C) and nitrogen (δ^15^N) isotopes, and C/N ratios in the oral arms, umbrella, and gonads of *C. andromeda* individuals, were assessed to better clarify the different nutrition behavior and growth processes of this species in the study area.

Small amounts (0.2 mg) of freeze-dried jellyfish tissue were prepared for the δ^13^C and δ^15^N analyses. The stable carbon and nitrogen isotopic signatures were determined using an isotope ratio mass spectrometer, DeltaV Advantage (Thermo Fisher Scientific, Bremen, Germany), coupled with a CHN Analyzer Flash 2000 (Thermo Fisher Scientific, Bremen, Germany). The analytical precision of the measurements for both δ^13^C and δ^15^N was 0.2%. Sucrose IAEA CH6 (International Atomic Energy Agency, Austria) and L-glutamic acid (RM 8574, National Institute of Standards and Technology, NIST, MA, USA) were used as certified reference materials.

The ratio of stable isotopes was expressed as delta (δ):

δ = [(Rsample/Rstandard) − 1)] × 10^3^,

where δ is the isotopic ratio of the sample relative to the standards (international standard: Vienna Pee Dee Belemnite for C, and atmospheric nitrogen for N); Rsample and Rstandard are the fractions of heavy to light isotopes in the sample and standard, respectively. This number is then multiplied by 1000 so that δ is expressed in units of parts per thousand (‰).

## 3. Results

**Figure 4:**
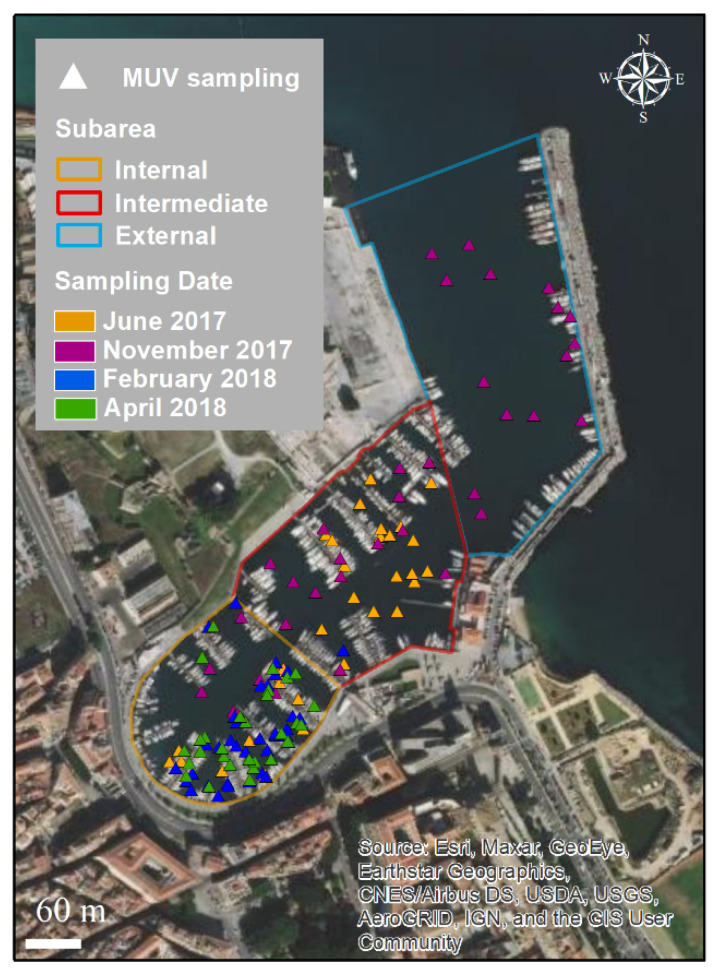
Sampling points per date using MUV within Cala.

A total of 146 points were sampled using Megabenthos Underwater Video (MUV) within Cala, divided as follows: 94 in the internal sub-area, 37 in the intermediate sub-area, and 15 in the external sub-area (Figure 4). The sampling depth ranged from 1.5 to 12 m. The water transparency ranged from 34 to 100%; values greater than 70% were recorded for 109 sampling points. Temperature and salinity ranged from 13.9 to 24.1 °C, and from 34.4 to 35.7, respectively.

### 3.1. Visual Data Analysis

Out of the 146 hauls, 39 were carried out in June, 38 were carried out in November, 39 were carried out in February, and 30 were carried out in April. A total of 67 individuals of *C. andromeda* were recorded, of which 30 belonged to the size class 5–10 cm, 34 to the size class 10–15 cm, and 3 to the size class 15–20 cm.

**Figure 5:**
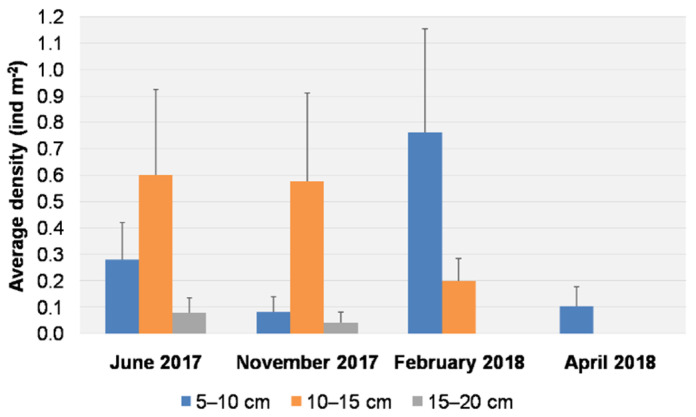
Average density (+S.E. as vertical bar) of *Cassiopea andromeda* individuals for each size class per sampling date.

The average densities for each size class per sampling date are reported in Figure 5. Individuals of the size class 5–10 cm were found on all dates, with a peak in density (0.76 ± 0.39 ind m^−2^) in February 2018, and were the only size class present in April 2018, with an average density of 0.10 ± 0.07 ind m^−2^. Individuals of the size class 10–15 cm were recorded in June 2017, November 2017, and February 2018, with higher average densities in June (0.60 ± 0.32 ind m^−2^) and November (0.57 ± 0.33 ind m^−2^). Larger individuals (15–20 cm) were observed only in June and November 2017, but at low densities (0.08 ± 0.05 ind m^−2^ and 0.04 ± 0.04 ind m^−2^, respectively).

**Figure 6:**
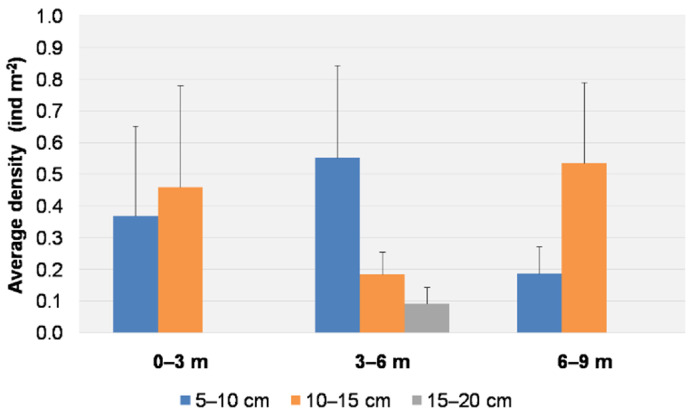
Average density (+S.E. as vertical bar) of *Cassiopea andromeda* individuals for each size class per depth range.

All individuals were found at depths of 1.5–7.5 m. All results are therefore reported for three ranges of depth (0–3, 3–6, 6–9 m). Individuals of the size 5–10 cm were found in all ranges, with a higher average density (0.55 ± 0.29 ind m^−2^) at depths of 3–6 m. Individuals of the size class 10–15 cm were found at all depths, with a higher average density (0.54 ± 0.25 ind m^−2^) at depths ranging from 6 to 9 m (Figure 6). Individuals of the size class 15–20 cm were found only between depths of 3 and 6 m, with a low average density (0.09 ± 0.05 ind m^−2^). The date and depth did not show any statistical influence on the size distribution (PERMANOVA, *p* > 0.05) (Table 1).

Out of the 67 individuals recorded, 52 and 15 were found in the internal and intermediate sub-areas, respectively. No individual was recorded in the external sub-area. The total average density was not significantly different between the internal (0.82 ± 0.25 ind m^−2^) and the intermediate (0.76 ± 0.35 ind m^−2^) sub-areas. The average depths (±standard deviation) of the sampling points were 5.4 ± 1.8 m in the internal sub-area, 7.3 ± 2 m in the intermediate sub-area, and 8.4 ± 1.7 m in the external sub-area. The sub-areas did not show any statistical influence on the size distribution (PERMANOVA, *p* > 0.05) (Table 1).

**Figure 7:**
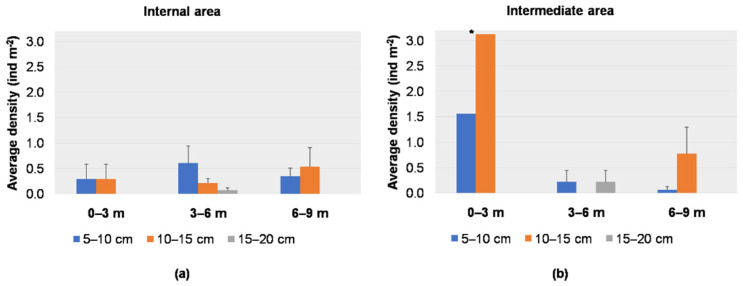
Average density (+S.E. as vertical bar) of *Cassiopea andromeda* individuals in the (**a**) internal sub-area and (**b**) intermediate sub-area; * one sampling point.

The average density for each size class per depth range for each sub-area is reported in Figure 7a,b. In the internal sub-area, the average density of small individuals (5–10 cm) was higher in the range 3–6 m (0.60 ± 0.34 ind m^−2^), while individuals of 10–15 cm were recorded more abundantly between 6 and 9 m depths (0.54 ± 0.37 ind m^−2^); larger individuals (15–20 cm) were recorded only in the range 3–6 m, with a low value (0.07 ± 0.05 ind m^−2^) (Figure 7a). In the intermediate sub-area, the higher average densities of individuals of 5–10 cm and 10−15 cm were recorded in the range 0–3 m (1.56 ind m^−2^ and 3.13 ind m^−2^, respectively); individuals of 15−20 cm were recorded only between 3 and 6 m depths (0.22 ± 0.22 ind m^−2^) (Figure 7b). Sub-areas and depth did not show any statistical influence on the size distribution (PERMANOVA, *p* > 0.05) (Table 1).

In Table 1, the results of the PERMANOVA on the average density of each size class per sampling date and depth range, per sub-area, and per depth range and sub-area are reported.

### 3.2. Spatial and Temporal Distributions

**Figure 8:**
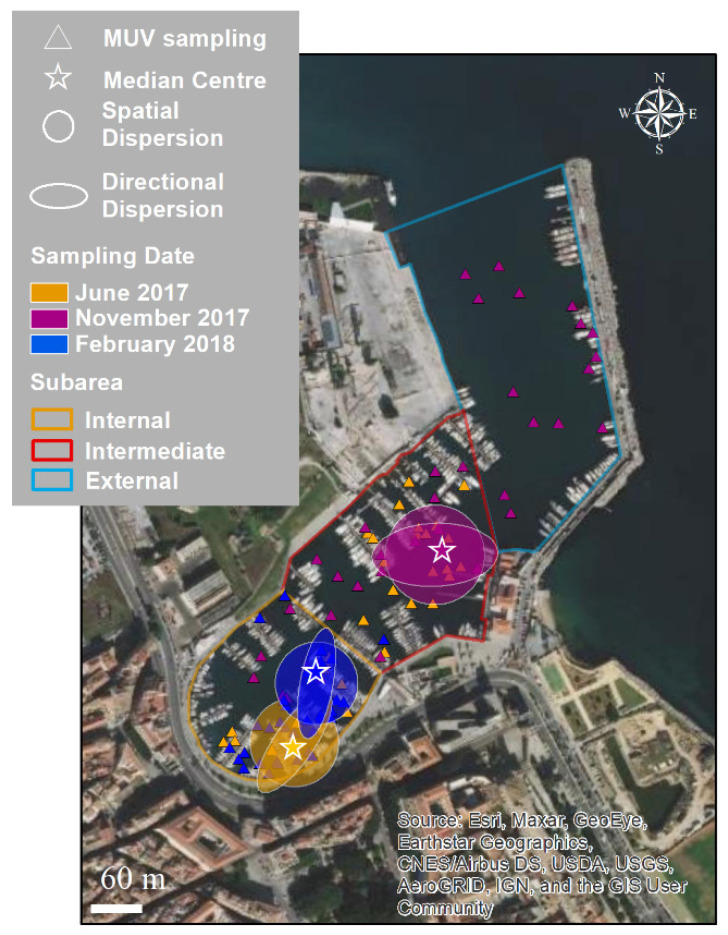
Key characteristics of the distribution of *Cassiopea andromeda* in Cala: central tendency (as a median center), spatial dispersion, and directional dispersion, calculated for June 2017, November 2018, and February 2018. The triangles indicate sampling points using MUV per date considered for the analysis.

In Figure 8, the values for central tendency, spatial dispersion, directional dispersion, and directional trend are mapped for each sampling date. The species’ spatial and directional dispersions were quantitatively similar in all periods but showed changes over time and space. In June, *C. andromeda* was primarily located in the internal sub-area, while in November, it was primarily located in the intermediate area. The directional trends were different in June and November, with angles of 31° and 83°, respectively.

**Figure 9:**
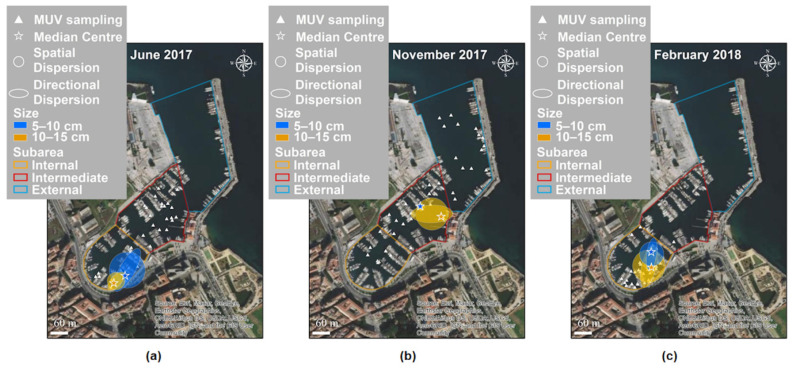
Key characteristics of the distribution of *Cassiopea andromeda* in Cala in (**a**) June 2017, (**b**) November 2018, and (**c**) February 2018, calculated for each size class. The central tendency (as a median center), spatial dispersion, and directional dispersion show changes in the distribution of size classes in space and time. The triangles indicate sampling points using MUV considered for the analysis.

Clear differences between the size classes can be seen in the key characteristics of the distributions per sampling date (Figure 9a–c). In June, the distribution of the 10–15 cm size class was more compact than that of the 5–10 cm size class. In November, the specimens observed mainly belonged to the 10–15 cm size class; their distribution was more dispersed than in June. In February, the 10–15 cm size class was more dispersed than the smallest size class.

Global spatial autocorrelations among the *C. andromeda* recorded in Cala were found for temperature, salinity, and transparency. The GMI values reported in Table 2 suggest that the distribution is clustered and non-random. Temperature and transparency showed greater spatial autocorrelation than salinity.

**Figure 10:**
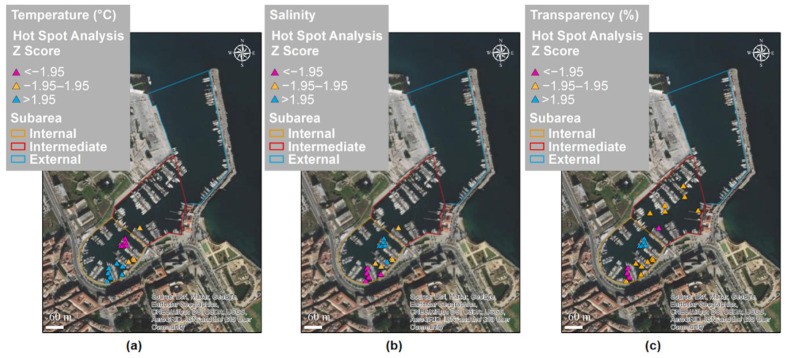
Results of the hot spot (Getis–Ord Gi*) analysis on *Cassiopea andromeda* records in Cala in relation to the environmental parameters (**a**) temperature, (**b**) salinity, and (**c**) transparency: blue and purple triangles indicate hot spots (high values) and cold spots (low values), respectively; yellow triangles indicate records with non-significant index values.

Figure 10a–c report the results of the hot spot (Getis–Ord Gi*) analysis on the MUV sampling of *C. andromeda* in relation to the environmental parameters temperature (T), salinity (S), and water transparency (Tr). At the local spatial scale, areas showing statistically significant clustering of low values (cold spot) and of high values (hot spot) were found. The other records (yellow triangles) had non-significant index values. In Table 3, the mean values ± standard error of the T, S, and Tr parameters corresponding to hot and cold spots are reported.

The spatial distributions of the three parameters for each sampling date are reported in Figures S1–S4.

### 3.3. Stable Isotope Analysis

**Figure 11:**
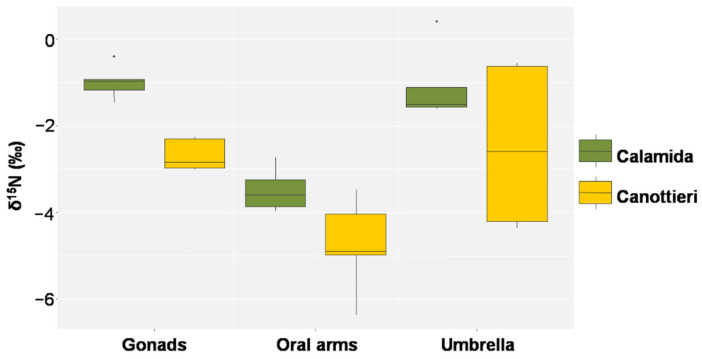
Box plot of δ^15^N in the gonads, oral arms, and umbrella of *Cassiopea andromeda* individuals collected at the two sites of Cala (*N* = 5 in Calamida and 5 in Canottieri).

**Figure 12:**
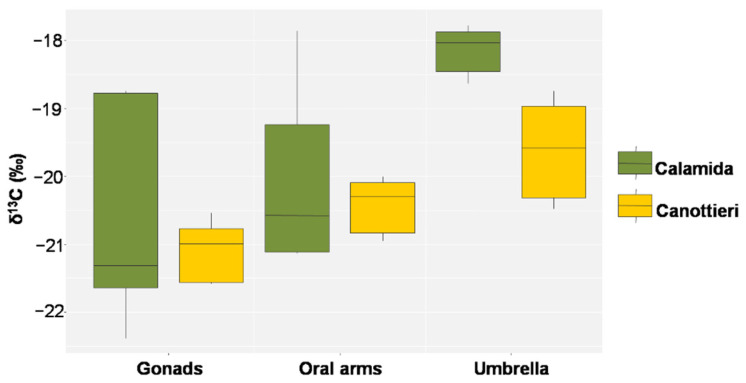
Box plot of δ^13^C in the gonads, oral arms, and umbrella of *Cassiopea andromeda* individuals collected at the two sites of Cala (*N* = 5 in Calamida and 5 in Canottieri).

**Figure 13:**
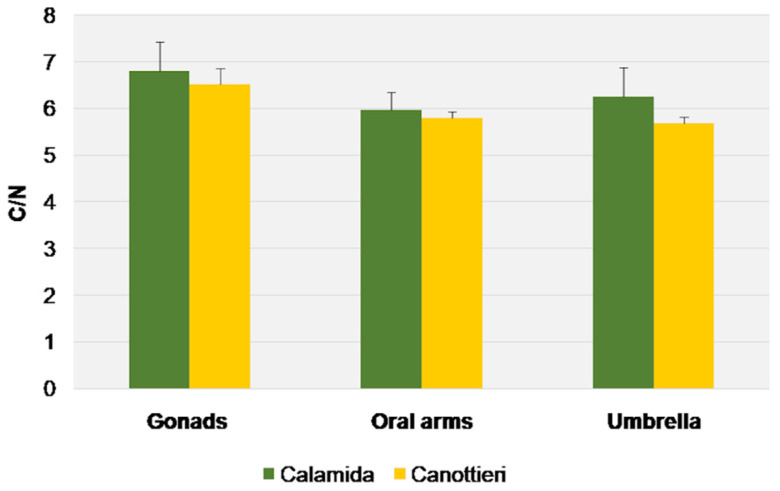
C/N molar ratio (average + S.E. as vertical bar) in the gonads, oral arms, and umbrella of *Cassiopea andromeda* individuals collected at the two sites of Cala.

In Figure 11, Figure 12 and Figure 13, the results of δ^15^N, δ^13^C, and the C/N molar ratio, respectively, found in the gonads, oral arms, and umbrella of the *C. andromeda* specimens sampled at both the “Calamida” and “Canottieri” sites are shown. The data reported for δ^15^N show negative values. For both sites, the values of δ^15^N were similar for the umbrella and gonads and lower for the oral arms (Δ 2.3‰ and 2.4‰ for Canottieri and Calamida, respectively) (Figure 11).

This evidence is also confirmed by the values of δ^13^C, which showed similar behaviors, despite the more pronounced differences in Calamida (Δ 0.8‰ and 1.8‰ for Canottieri and Calamida, respectively). The more negative δ^13^C and higher C/N ratio values observed for the gonads could depend on the higher lipid content in this tissue (Figure 12 and Figure 13).

**Figure 14:**
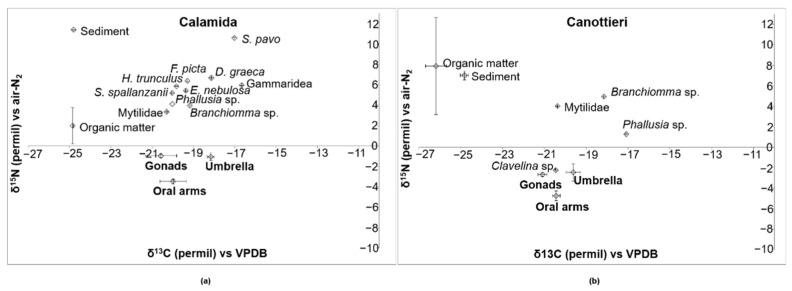
Bidimensional plot of δ^15^N versus δ^13^C (average ± S.E. as vertical bar) in the gonads, oral arms, and umbrella of *Cassiopea andromeda* individuals; sediments, organic matter, and some benthic organisms (*Branchiomma* sp.; *Clavelina* sp.; *Diodora graeca*; *Eupolymnia nebulosa*; *Felimare picta*; Gammaridea; *Hexaplex trunculus*; Mytilidae; *Phallusia* sp.; *Salaria pavo*; and *Sabella spallanzanii*) collected from two sites of Cala: (**a**) Calamida and (**b**) Canottieri.

Figure 14a,b show bidimensional plots of δ^15^N and δ^13^C in three body parts of *C. andromeda* and the other community components sampled at the Calamida and Canottieri sites. The values of the two stable isotopes in *C. andromeda* show similar trends for the two sites, showing less negative values in the umbrella than in the oral arms. The Calamida site probably reflects a less disturbed situation, showing a shift from lower to higher values along the trophic chain for the two isotopes. Specifically, along with the three body parts of the jellyfish and the sediment (δ^15^N = 11.37‰ and δ^13^C = −24.79‰—one sample), Figure 14a shows three main groups (average ± S.E. of δ^15^N and δ^13^C): (1) organic matter (δ^15^N = 1.96 ± 1.79‰ and δ^13^C = −24.85 ± 0.01‰); (2) filter feeders/detritivores: *Branchiomma* sp. Anellida, *Eupolymnia nebulosi* Anellida, Mytilidae, *Phallusia* sp. Tunicata, and *Sabella spallanzanii* Anellida (δ^15^N = 4.39 ± 0.38‰ and δ^13^C = −19.77 ± 0.21‰); and (3) predators: *Diodora graeca* Mollusca, *Felimare picta* Mollusca, *Hexaplex trunculus* Mollusca, Gammaridea Crustacea, and *Salaria pavo* Osteichthyes (δ^15^N = 7.08 ± 0.88‰ and δ^13^C = −18.18 ± 0.61‰). The situation at the Canottieri site is different; even though the filter feeders/detritivores (*Branchiomma* sp., Mytilidae, *Phallusia* sp., and *Clavelina* sp. Tunicata) present similar values (δ^15^N = 1.99 ± 1.61‰ and δ^13^C = −19.01 ± 0.83‰) to those at the Calamida site, the organic matter (δ^15^N = 7.91 ± 4.72‰ and δ^13^C = −26.18 ± 0.50‰) and the sediments (δ^15^N = 7.00 ± 0.42‰ and δ^13^C = −24.81 ± 0.21‰) have very different values compared with other community components.

## 4. Discussion

Non-indigenous species (NIS) have attracted the attention of researchers due to the impacts they can have on biodiversity, the economy, and human health. Harbors and marinas are among the main hotspots for the introduction of alien species, due to maritime traffic being intense [49,50]. NIS monitoring and research are essential in improving the knowledge on species invasiveness, in evaluating their potential threat to the surrounding ecosystem, and in supporting management actions. One such alien species is the upside-down jellyfish *Cassiopea andromeda*, the invasion of which has been recently reported in a Mediterranean touristic harbor of Palermo (Italy): “Cala” [5].

### 4.1. Distribution of Cassiopea Andromeda

Studying NIS in harbor environments is not always easy due to the presence of obstacles (e.g., floating docks, anchored or moving nautical vehicles, and ropes) which hinder the use of standard visual methods and tools. A Megabenthos Underwater Video (MUV) device specially designed and built to overcome these sampling difficulties [40] allowed recording several *C. andromeda* specimens of different sizes in the different sub-areas of Cala. Their abundance and density varied across the four sampling dates. The large number of individuals of small sizes observed in February 2018 compared with that during previous months, when the intermediate size was the most abundant, suggests a previous reproductive event and indicates that the population of jellyfish in this study area is quite established.

The lowest values of abundance and density were recorded in April 2018, when the medusa stage population was clearly in decline. Since then, the medusa stage disappeared for two years and was subsequently spotted in October 2020 and in November 2021 (unpublished data from the authors’ on-site visual inspections), when a few small and medium-sized specimens were observed. The period of apparent absence of the species in this harbor of Palermo could correspond to the polyp phase, which is not macroscopically visible. After a long period of two years, the transition from the polyp to medusa (strobilation) phases could have been triggered by exogenous factors (according to [11]), which led to the reappearance of a visible population.

The presence of *C. andromeda* down to 7.5 m depths may be due to the jellyfish’s photosynthetic symbionts needing light, and its presence in the internal and intermediate zones of Cala could be due to the more eutrophic conditions [8]. Moreover, the dense packing of the smallest individuals in the internal zone could depend on the hypothetical presence of a close polyp population due to the lower hydrodynamics and the presence of many artificial substrates in this area of the harbor. Equally, the lack of *C. andromeda* observed at deeper and external sites (i.e., at the Cala mouth) may also be due to the greater amount of water movement typically caused by the continuous passage of boats.

From the results of the GIS-based statistical analyses, the jellyfish aggregation seen during June and November in different sub-areas and with different orientations suggests that the *C. andromeda* population is established in various zones of Cala. This is probably due to the environmental variability of the harbor, which is severely influenced by various factors (e.g., terrigenous and anthropogenic inputs) heterogeneously distributed over time and space, as well as by the variation in some environmental parameters (e.g., water turbidity due to vessel movements and external inputs).

The ranges of the environmental parameters measured in the study area (temperature, salinity, and transparency) were consistent with the conditions necessary to maintain a *Cassiopean* population [37] and did not influence the distribution of specimens, which clustered around both low and high parameter values. In fact, *Cassiopea* spp. tolerate a wide range of temperatures (up to about 29 °C), salinities (up to 36), and levels of light exposure (from 200 to 500 μmol photons m^−1^ s^−1^) [10,38]. The cluster behavior is possibly generated by other factors, such as a greater concentration of nutrients or organic matter in these areas, which would attract jellyfish to specific areas, as hypothesized by [37].

### 4.2. Trophic Behavior of Cassiopea Andromeda

In the literature, the use of δ^15^N from aquatic autotrophic organisms and consumers to trace anthropogenic sources of N has often been reported [51,52,53,54,55]. The generally most useful observation is that, in nutrient-rich environments, the δ^15^N of algae can track the δ^15^N from nitrate. The transformation of inorganic nitrogen compounds into an organic form during biosynthesis by living autotrophic organisms influences the reduction of oxidized forms of N to ${\mathrm{NH}}_{4}^{+}$ and, then, its assimilation into organic matter. This process generally prefers the incorporation of an isotope with a lower mass. Some authors [56,57] measured a large range of N fractionations (−30 to 0‰) for nitrate and ammonium assimilation by algae and bacteria. The atmospheric N_2_ bacterial fixation by the enzyme nitrogenase is reflected in organic material having δ^15^N values slightly less than 0‰ and lower than the environmental values for organic materials produced by other mechanisms [58]. For this reason, low δ^15^N values in organic matter are generally thought to indicate N_2_ fixation. Assimilation produces isotope fractionation by favoring the incorporation of lighter isotopes. Several authors measured a wide range of N fractionations (−30 to 0‰) in field studies [56,57] and in laboratory experiments for nitrate and ammonium assimilation by algae [59,60,61]. For ammonium assimilation, the authors of [58] reported a range of fractionation from −4 to −27‰, depending on whether the algae cells were nitrogen limited, enzyme limited, or diffusion limited.

The *Cassiopea andromeda* trophic position and energy assimilation strategy may vary according to the availability of food sources, allowing the species to subsist in water masses ranging from eutrophic to oligotrophic conditions [62,63,64].

In the study area, the lower values of δ^15^N for *C. andromeda*, indicating a depletion of ^15^N compared with ^14^N, were unexpected, but they could be the result of the availability of nitrogen sources rich in ^14^N, such as those from untreated urban waste [65]. These lower values could be more affected by the characteristic of the sampling sites (Calamida and Canottieri), which are confined and feature low hydrodynamics. The stable isotope values in *C. andromeda* were consistently lower than those in the other community components, suggesting the production of organic components based on the metabolism of associated symbionts.

Septic tank effluent contains predominantly organic and inorganic carbon, organic nitrogen, and ammonium. The septic sludge δ^15^N reported by [65] had low values (–2.1‰), whereas the corresponding particulate and dissolved fractions generally had different nitrogen and carbon ratios as a result of fractionation in the anaerobic or aerobic processes. Nevertheless, an influence of atmospheric precipitation was not excluded given that the δ^15^N of nitrate reported for a wet deposition showed values ranging from −11‰ to +3.5‰, with a mean value of −3.1‰ [66,67,68].

For both sampling sites, the lower δ^15^N values observed for the oral arms of jellyfish suggest a lower trophic level with respect to the umbrella, highlighting the higher concentration of autotrophic symbionts in these tissues [11,69]. Indeed, the pattern suggested is that the zooxanthellae, which colonize the oral arms of *C. andromeda*, uptake the dissolved inorganic nitrogen with a low δ^15^N value from the environment [63,64,70], while the umbrella uses a mix of the uptake of nitrogen with higher δ^15^N values, due to fractionation along the food web through predation [24,62,71], and degradation of the colonizers.

Less negative δ^13^C values (typically from −10‰ to −14‰) than those of particulate organic matter and plankton (ca. −20‰ [62]) are typical for the uptakes of dissolved inorganic carbon by zooxanthellae [20,22,72,73]. The δ^13^C values obtained for the umbrella in this study were higher than those obtained for the oral arms, in particular at the Calamida site, suggesting less translocation of the metabolites derived from symbionts and greater dependence on the heterotrophic metabolism of the jellyfish [63,64].

During synthesis, the isotopic signature evidenced that the metabolites derived from inorganic nutrient uptake and predation are then exchanged and recycled between the zooxanthellae and the host (e.g., [74]). The photosynthesis that occurs in the oral arms could involve processes depleting the nutrient pools derived from untreated discharge civil effluents and mixed with the discharge of wet depositions.

## 5. Conclusions

In the touristic harbor of Palermo, “Cala”, the *Cassiopea andromeda* population showed alternating periods of abundance, predominantly the medusa stage, and periods of apparent quiescence. The specimens showed adaptation to both winter and summer temperatures, settings found in the shallow bottoms of the more confined areas of Cala. However, further investigations on the species distribution, including other environmental parameters (such as nutrients and light exposure) and seasonal differences, are still needed to better understand how the species established itself and clustered within the study area.

*C. andromeda* showed a dual nutrition strategy (mixotrophic behavior) with stable isotope values that were consistently lower than those in the other community components, probably due to the presence of N derived from human sources. However, additional studies on the nutrient dynamics in this area and the isotopic values of other community components (phyto- and zooplankton) would help to clarify the trophic role of this species in the environment.

All preliminary results confirm that *C. andromeda* is capable of living and reproducing in heavily anthropized areas such as harbors, allowing the species to establish itself once introduced and to be transferred elsewhere by boats [10]. Moreover, the species seems to have the ability to cope with environmental changes. These traits, together with its mixotrophy, render the species a perfect invader in current and future scenarios of climate change. Its potential impact on the local biodiversity and thus on the marine ecosystem’s structure and functioning is worth considering. Therefore, specific surveillance actions, performed by trained staff, would allow for monitoring and control of these invasions, which may be a threat to the biodiversity and ecosystems of a certain region.

## Figures and Tables

**Table 1:** Results of the PERMANOVA on the average density of each size class: per sampling date (SD) and depth range (DR), per sub-area (SA), and per depth range and sub-area.

Source	d.f.	MS	Pseudo-F	*p*-Value
SD	3	0.51	0.764	0.57
DR	3	0.39	0.586	0.73
SD × DR	8	0.47	0.699	0.75
SA	2	0.88	1.342	0.24
DR	3	0.25	0.388	0.76
SA	2	0.12	0.193	0.78
DR × SA	3	0.08	0.133	0.93

**Table 2:** Spatial autocorrelation using Global Moran’s I (GMI) for the *Cassiopea andromeda* distribution in Cala performed on temperature (°C), salinity, and transparency (%). GMI expected index, variance, z-score, and *p*-value are also reported.

	Moran’s Index	Expected Index	Variance	z-Score	*p*-Value
Temperature (°C)	0.89	−0.02	0.006	>2.58	<0.01
Salinity	0.33	−0.02	0.006	>2.58	<0.01
Transparency (%)	0.71	−0.02	0.005	>2.58	<0.01

**Table 3:** Average ± S.E. for temperature, salinity, and transparency corresponding to hot and cold spots, with the z-score and *p*-value.

	Cold Spot	Hot Spot	z-Score	*p*-Value
Temperature (°C)	14.86 ± 1.96	21.52 ± 3.30	>1.96	<0.01
Salinity	35.07 ± 0.14	35.28 ± 0.16	>1.96	<0.01
Transparency (%)	93 ± 13	100 ± 0	>1.96	<0.01

## Data Availability

Data sharing not applicable.

## Supplementary Materials

The following supporting information can be downloaded at: https://www.mdpi.com/article/10.3390/biology11020319/s1, Figure S1: Spatial distribution of (a) temperature (°C), (b) salinity, and (c) transparency (%) in June 2017. The white triangles indicate the measurement points of the parameters; Figure S2: Spatial distribution of transparency (%) in November 2017. The white triangles indicate the measurement points of the parameter; Figure S3: Spatial distribution of (a) temperature (°C), (b) salinity, and (c) transparency (%) in February 2018. The white triangles indicate the measurement points of the parameters; Figure S4: Spatial distribution of (a) temperature (°C), (b) salinity, and (c) transparency (%) in April 2018. The white triangles indicate the measurement points of the parameters.
