# Supplementary material for: Snapshot of the Distribution and Biology of Alien Jellyfish Cassiopea andromeda (Forsskål, 1775) in a Mediterranean Touristic Harbour
Source: Biology (Basel). 2022 Feb 16;11(2):319. doi: 10.3390/biology11020319 (PMC8868670; doi:10.3390/biology11020319)
Supplement: Supplementary file 1 [file biology-11-00319-s001.zip › biology-1512994-SI.pdf]

Cillari T., Allegra A., Berto D., Bosch-Belmar M., Falautano M., Maggio T., Milisenda G., Perzia P., Rampazzo F., Sinopoli M. and Castriota L.: “*Snapshot of the distribution and biology of alien jellyfish Cassiopea andromeda (Forsskål, 1775) in a Mediterranean touristic harbour*”
- Supplementary materials

In the touristic harbour of Palermo (Italy) named “Cala”, visual samplings were carried using Megabenthos Underwater Video (MUV) [40] at three sub-areas (internal, intermediate, and external) and on four date (June 2017, November 2017, February 2018, and April 2018). At each sampling point, the temperature (°C) and salinity along the water column were recorded, using a portable meter (VWR EC300), and water transparency (m) recorded using a Secchi disc. The latter parameter was reported as percentage ratio between water transparency (m) and depth (m).
The temperature (T), salinity (S), and transparency (Tr) dataset was processed using ArcGIS 10.3 ESRI and its Spatial Analyst toolbox (i.e. Topo to Raster tool in Raster toolset Interpolation) to obtain the spatial distribution of the parameters [47]. The polygons related to the three sub-areas were used as spatial constraints for the interpolation process. T (°C), S, and Tr (%) spatial distributions were mapped for each sampling date (Figures S1,S2,S3,S4).

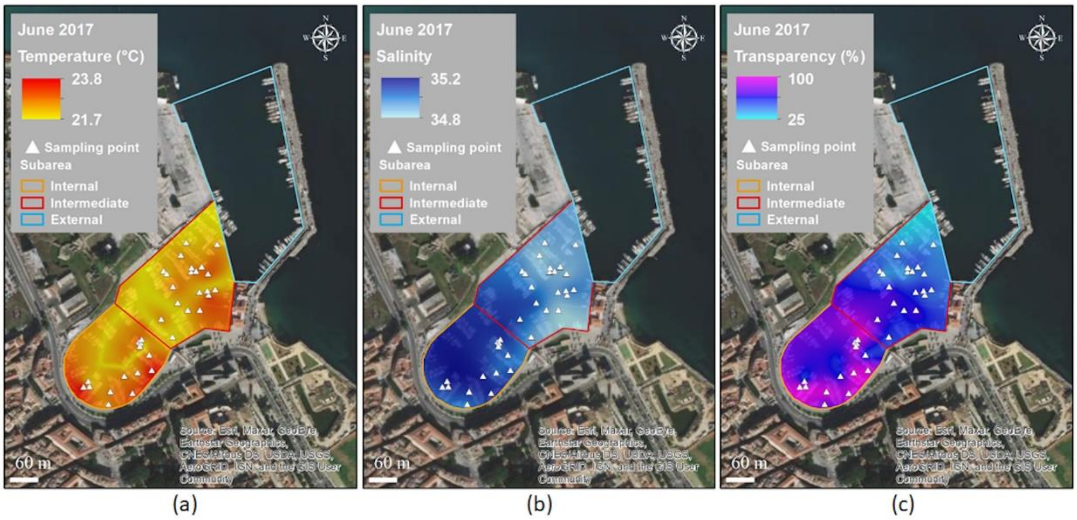

Figure S1: Spatial distribution of (**a**) temperature (°C), (**b**) salinity, and (**c**) transparency (%) in June 2017. The white triangles indicate the measurement points of the parameters.

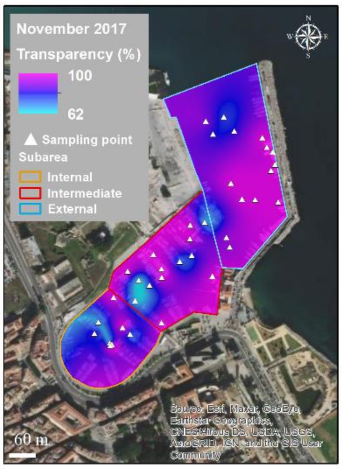

Figure S2: Spatial distribution of transparency (%) in November 2017. The white triangles indicate the measurement points of the parameter.

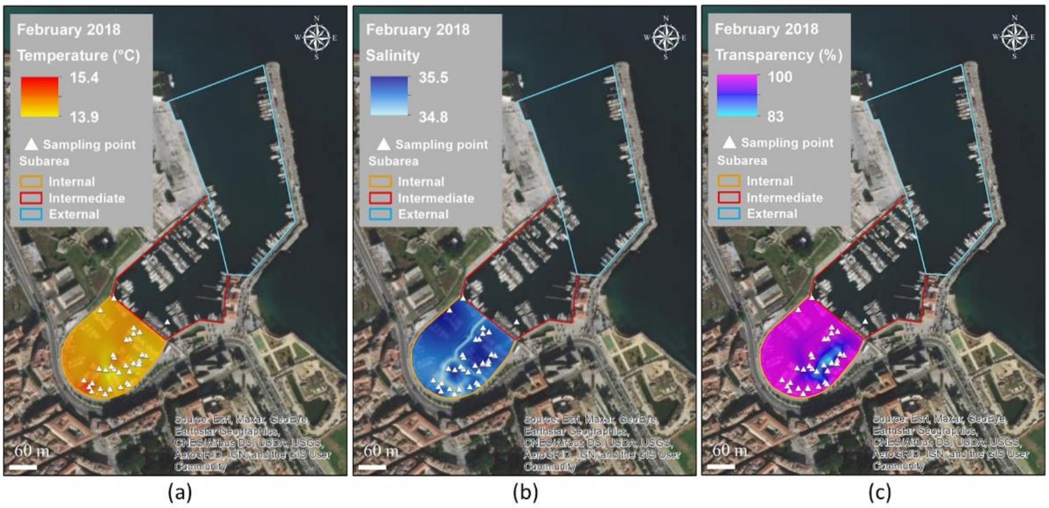

Figure S3: Spatial distribution of (**a**) temperature (°C), (**b**) salinity, and (**c**) transparency (%) in February 2018. The white triangles indicate the measurement points of the parameters.

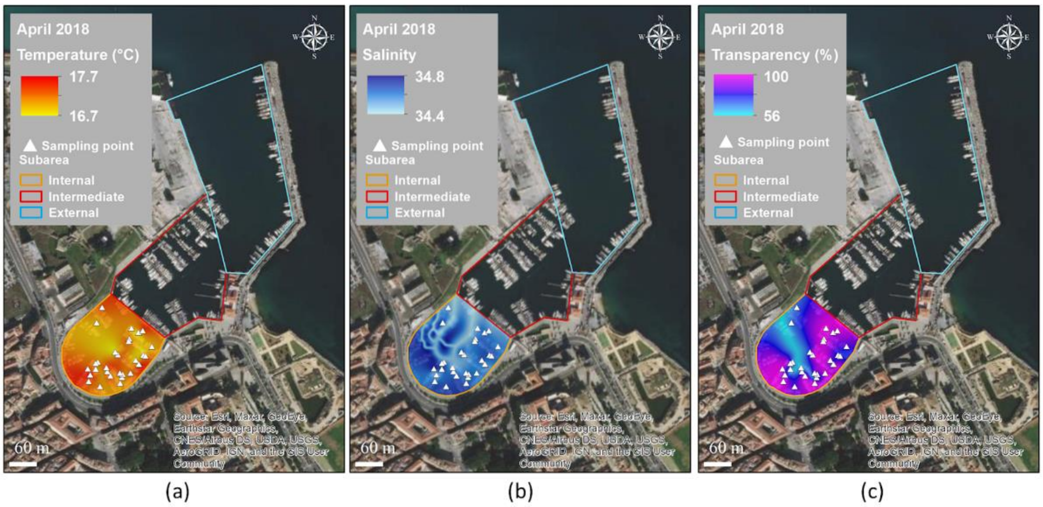

Figure S4: Spatial distribution of (**a**) temperature (°C), (**b**) salinity, and (**c**) transparency (%) in April 2018. The white triangles indicate the measurement points of the parameters.
